# Matrix Metalloproteinase 14 in Corneal Neovascularization

**DOI:** 10.3390/ijms27042027

**Published:** 2026-02-20

**Authors:** Kaley Qin, Nicholas W. Setter, Lily Yu, Zahra Vasi, Weiyu Wu, Yunjeong Hwang, Hyun Lee, Jin-Hong Chang, Mark I. Rosenblatt, Kyuyeon Han, Dimitri T. Azar

**Affiliations:** 1 Department of Ophthalmology and Visual Sciences, Illinois Eye and Ear Infirmary, College of Medicine, University of Illinois Chicago, Chicago, IL 60612, USA; kaleyq2@uic.edu (K.Q.); settern@uic.edu (N.W.S.); lyu39@uic.edu (L.Y.); zvasi2@uic.edu (Z.V.); wwu74@uic.edu (W.W.); yjhwang1@uic.edu (Y.H.); changr@uic.edu (J.-H.C.); mrosenbl@uic.edu (M.I.R.); 2 Department of Pharmaceutical Sciences, College of Pharmacy and Biophysics Core, the Research Resource Center, University of Illinois at Chicago, Chicago, IL 60607, USA; danielhl@uic.edu; 3 Jesse Brown Veterans Affairs Medical Center, Chicago, IL 60612, USA

**Keywords:** MMP-14, metalloproteinase, corneal neovascularization, corneal angiogenesis, VEGF signaling, corneal wound healing, TIMP-2, matrix remodeling

## Abstract

Corneal neovascularization (CoNV) disrupts the natural avascularity of the cornea, leading to loss of transparency and visual impairment. Among matrix metalloproteinases (MMP), MMP-14, a membrane-bound MMP, plays a central role in CoNV through matrix remodeling, activation of pro-MMP-2, modulation of growth factors-induced signaling, and regulation of vascular endothelial cell behavior. Under pathogenic conditions, MMP-14 promotes angiogenesis by degrading stromal collagen, enhancing vascular endothelial growth factor (VEGF) signaling, and stimulating vascular endothelial cell migration. However, MMP-14 can also exert anti-angiogenic effects by generating endostatin-like fragments such as neostatin-14. MMP-14 also participates in corneal wound healing and lymphangiogenesis, making it a promising therapeutic target for CoNV. Standard therapies for CoNV, such as corticosteroids, immunosuppressants, and anti-VEGF agents, remain partially effective. Novel strategies targeting MMP-14, including small-molecule inhibitors, selective use of TIMP-2, and recombinant antibodies, are being explored. A deeper understanding of how membrane-bound MMP-14 is regulated and functions in different contexts may allow better modulation of angiogenesis, ultimately preserving corneal clarity and visual function after injury or inflammation.

## 1. Introduction

Matrix metalloproteinases (MMP) participate in several physiologic and pathologic processes [1] and can exert both pro- and anti-angiogenic activity [2,3]. Among them, MMP-14 is prominently upregulated during neovascularization and plays a major role in orchestrating the angiogenic response. As a membrane-anchored metalloproteinase, MMP-14 is strategically positioned at the cell surface, where it not only cleaves extracellular matrix (ECM) components, such as collagen type I [4] and activates pro-MMP-2 to enable migration of vascular endothelial cells (VECs) and tubulogenic organization within the stromal matrix [5], but also interacts more readily with other membrane-associated proteins and receptors. This capability that distinguishes it from soluble or secreted MMPs and allows it to exert direct, localized control over angiogenic signaling events [6].

**Figure 1:**
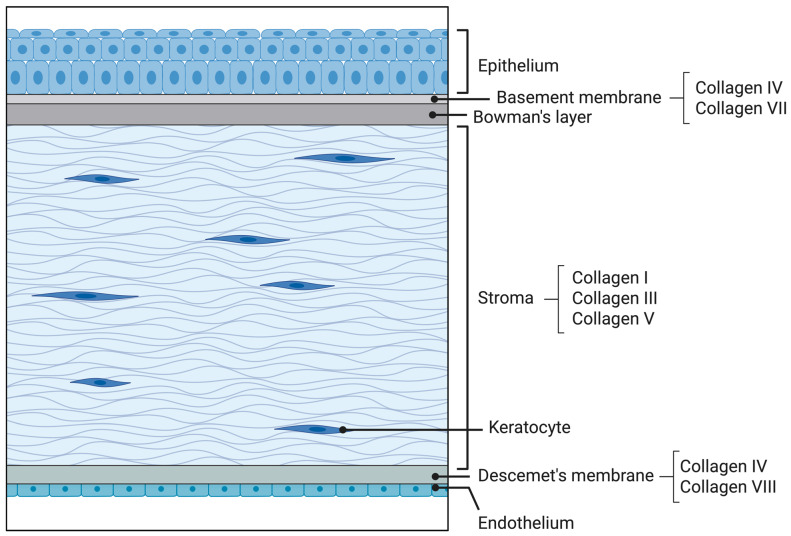
Normal corneal architecture and collagen distribution. Schematic representation of the cornea illustrating the epithelium, epithelial basement membrane, Bowman’s layer, stroma (keratocytes), Descemet’s membrane, and endothelium. Key collagen types are indicated, with collagens I, III, and V in the stromal extracellular matrix and collagens IV and VII in the epithelial basement membrane.

Corneal avascularity is critical for maintaining optical transparency and ensuring visual performance. This “angiogenic privilege” is an active state sustained by a delicate equilibrium in which anti-angiogenic factors counterbalance pro-angiogenic mediators [7]. However, various ocular injuries can disrupt corneal homeostasis and compromise this privilege by inducing inflammation and elevating the production of pro-angiogenic factors [8]. The resulting upregulation of pro-angiogenic signals and concurrent reduction in anti-angiogenic factors allows capillaries from the limbal plexus to invade the normally avascular stroma, ultimately leading to corneal neovascularization (CoNV) [8]. For anatomical context, Figure 1 depicts the normal corneal layers and the distribution of key collagen types.

The formation of new vessels follows a tightly regulated stepwise sequence [9]. After corneal injury or inflammation, resident cells release pro-angiogenic factors, including vascular endothelial growth factor (VEGF), that activate VECs [10]. Once activated, VECs initiate the angiogenic program by upregulating proteases, degrading their basement membrane and the adjacent ECM, and adopting a migratory tip-cell phenotype. Guided by chemotactic gradients and matrix cues, these VECs invade the corneal stroma, proliferate to form multicellular endothelial cords, and subsequently undergo lumen formation and branching to establish new vascular networks [10,11]. These nascent vessels are structurally immature and highly permeable, allowing stromal fluid leakage that leads to edema, loss of transparency, and disruption of corneal architecture and function [10]. MMP-14 plays an important role in ECM degradation, stromal invasion, and lumen formation. The following sections progressively narrow from general MMP biology to the specific and context-dependent roles of MMP-14 in corneal neovascularization.

## 2. Matrix Metalloproteinases

### 2.1. General Biochemical and Biological Attributes of MMPs

MMPs are a family of zinc-dependent endopeptidases that mediate ECM degradation and tissue remodeling. Their catalytic mechanism, which relies on a zinc ion coordinated within the active site, places them within the broader class of metalloproteases and specifically within the metzincins superfamily [12]. MMPs participate in diverse biological processes by degrading structural ECM components, regulating cell–matrix and cell–cell interactions, and modulating the bioavailability of growth factors and signaling molecules. Through this activation, they influence tissue remodeling, wound healing, angiogenesis, and inflammatory responses [1]. Beyond matrix substrates, MMPs also process cytokines, chemokines, and other signaling proteins, contributing to immune-cell recruitment and orchestration of inflammation [13]. Here, we provide a brief overview of MMP biology for context. MMP-14–specific structure–function features are discussed in Section 3, and therapeutic considerations including inhibition strategies are addressed in Section 6.

MMPs were first recognized for their collagenolytic activity following the discovery of tadpole collagenase in 1962 [14], establishing their fundamental roles in ECM degradation [15]. Since then, 28 human MMPs have been identified, each with distinct tissue distributions and biological functions. Collectively, these enzymes participate in key cellular processes such as cell proliferation, migration, and differentiation [16], and their dysregulation contributes to numerous pathological conditions, highlighting their relevance as potential therapeutic targets [17]. Despite their functional diversity, MMPs share a highly conserved molecular structure consisting of a propeptide domain (≈80 aa), a catalytic metalloproteinase domain containing a zinc-binding site (≈170 aa), a hinge region of variable length, and a hemopexin domain (≈200 aa), except in the matrilysins, which lack the hemopexin domain. Additional defining features include homology to collagenase-1 (MMP-1), a cysteine-switch motif that maintains the enzyme in its latent pro-MMP form by coordinating the catalytic zinc ion, and a conserved zinc-binding motif within the catalytic domain [1]. MMPs’ activity is primarily regulated at the level of gene expression and proenzyme activation, and is further modulated by growth factors, hormones, cytokines, and other extracellular cues [1].

MMPs are categorized based on their structure and substrate specificity into six major groups: collagenases, gelatinases, stromelysins, matrilysins, membrane-type (MT-MMPs), and a miscellaneous group comprising other MMPs [1]. Collagenases degrade fibrillar collagens, whereas gelatinases target denatured collagen (gelatin), among other ECM components. Stromelysins degrade a broad range of ECM substrates but not fibrillar collagens. Matrilysins are notable for lacking the hemopexin domain [1]. Membrane-Type MMPs, including MMP-14, contain a transmembrane domain or glycosylphosphatidylinositol (GPI) anchor that localizes them to the cell surface, enabling pericellular proteolysis and the activation of other MMPs, including pro-MMP-2. The remaining MMPs comprise diverse members with various substrates and biological roles. Additional information on the distribution, substrate specificity, and functional significance of each MMP is provided in Supplementary Table S1.

### 2.2. The Role of Matrix Metalloproteinases in Ocular Disease

Alterations in the ECM composition and turnover are central to the development and progression of many ocular diseases [18]. Because MMPs and their endogenous inhibitors, the tissue inhibitors of metalloproteinases (TIMPs), are key regulators of ECM remodeling, dysregulation of their expression or activity is closely linked to disease onset, progression, and tissue response to injury. Disruption of the MMP/TIMP balance can compromise tissue homeostasis, promote pathological remodeling, and contribute to a broad spectrum of ocular disorders affecting both the anterior and posterior segments of the eyes [18].

MMPs are implicated in multiple ocular pathologies, including ocular surface disease, glaucoma, retinal vascular disorders, and ocular malignancies. In ocular cancer—particularly retinoblastoma—elevated levels of MMP-1, MMP-2, and MMP-9 correlate with increased tumor severity, consistent with the established role of gelatinases in tumor invasion and metastasis [18,19]. Beyond malignancy, aberrant MMP activity are associated with cataracts, primary open-angle glaucoma, diabetic retinopathy, dry eye disease (DED), and age-related macular degeneration (AMD) [18].

In primary open-angle glaucoma, MMP-2 and MMP-9 are expressed within the trabecular meshwork and contribute to ECM remodeling, a process implicated in changes in aqueous humor outflow resistance [18,20,21]. In diabetic retinopathy, increased activity of MMP-2, MMP-9, and MMP-14 contributes to blood–retinal barrier breakdown and enhanced vascular permeability, linking MMP dysregulation to microvascular dysfunction [22]. In AMD, MMPs are implicated in the degradation of Bruch’s membrane and the facilitation of choroidal neovascularization, connecting abnormal ECM remodeling to both atrophic and neovascular disease phenotypes [23].

On the ocular surface, MMP-9 plays a particularly prominent role in DED. Elevated tear concentrations of MMP-9 are associated with increased disease severity, reduced tear breakup time, and compromised corneal epithelial integrity [24]. Pro-inflammatory cytokines such as IL-1β and tumor necrosis factor alpha (TNF-α) induce MMP-9 expression [25,26], leading to degradation of epithelial tight-junction proteins, barrier disruption, and accelerated epithelial cell shedding [27]. This process establishes a self-amplifying inflammatory loop that exacerbates ocular surface damage, positioning MMP-9 as both a mediator and biomarker of disease activity. Clinically, MMP-9 detection has been translated into tear-based point-of-care assays [28] and is highlighted as a biomarker in the most recent Tear Film & Ocular Surface Society Dry Eye Workshop (TFOS DEWS III) [29].

In infectious keratitis, excessive collagenolytic activity driven by MMPs plays a central role in disease pathophysiology. MMP-2 and MMP-9 are markedly upregulated and contribute directly to stromal collagen degradation, ulcer formation, and corneal thinning [30]. An imbalance between multiple MMPs—including MMP-1, MMP-2, MMP-8, and MMP-9—and their inhibitors disrupts corneal homeostasis and promotes excessive tissue destruction in bacterial, herpetic, fungal, and Acanthamoeba keratitis [31].

MMPs also contribute to the development and progression of pterygium, a fibrovascular ocular surface lesion characterized by aberrant ECM remodeling [32]. Increased expression of MMP-1, MMP-2, MMP-3, MMP-7, MMP-8, and MMP-9 has been reported in pterygium tissue [33,34], with particularly strong expression of MMP-2 and MMP-9 in pterygium-derived fibroblasts during lesion progression [35]. As a fibrotic disorder, pterygium is strongly regulated by transforming growth factor-β1 (TGF-β1) signaling [36]. Integrin αvβ8–mediated activation of latent TGF-β1 stimulates MMP-14 expression [37], which contributes to collagen degradation, fibroblast migration, and sustained fibrotic remodeling within the lesion [32].

Although numerous experimental and clinical studies support associations between specific MMPs and ocular diseases, the precise regulatory pathways governing MMP activity in each condition remain incompletely understood [18]. In addition, while MMPs and TIMPs represent promising therapeutic targets, clinical translation has been limited by insufficient mechanistic clarity and challenges in achieving selective modulation. Continued investigation into the roles of individual MMPs and the MMP/TIMP balance may reveal critical insights into ocular pathogenesis and inform the development of targeted therapies.

## 3. MMP-14 Background

### 3.1. Structural and Biochemical Features

MMP-14 (also known as MT1-MMP) is the prototype of the membrane-type matrix metalloproteinase family and is encoded on human chromosome 14q11-q12 [18]. Like other MT-MMPs (MMP-15, -16, -17, -24, and -25), MMP-14 is synthesized as an inactive zymogen, activated intracellularly, and subsequently transported to the plasma membrane, where it functions as a membrane-anchoring endopeptidase [1]. Its domain architecture includes an N-terminal propeptide, a catalytic metalloproteinase domain containing a zinc-binding motif, a flexible hinge region, and a C-terminal hemopexin domain, followed by a transmembrane region and a short cytoplasmic tail [38]. As a zinc- and calcium-dependent protease, MMP-14 preferentially cleaves substrates containing small or medium-sized hydrophobic residues at the P1′ position [17]. It exhibits potent collagenolytic activity, degrading fibrillar collagen type I, II, and III into the characteristic 3/4 and 1/4 fragments [4]. This activity is enhanced through functional cooperation with MMP-2, reflecting the well-established synergy between MMP-14–mediated collagen cleavage and pro-MMP-2 activation [4,39]. In addition to collagens, MMP-14 degrades a broad range of extracellular matrix components, including fibronectin and laminin, underscoring its central role in extracellular matrix remodeling [17,18].

Beyond direct matrix degradation, MMP-14 serves as the principal cell-surface activator of pro-MMP-2 through the formation of a TIMP-2–dependent trimolecular complex (MMP-14/TIMP-2/proMMP-2) [40,41]. TIMP-2 binds the catalytic site of one MMP-14 molecule, leaving an exposed domain of TIMP-2 to interact with proMMP-2. To activate proMMP-2, a neighboring MMP-14 molecule, free of TIMP-2, cleaves the TIMP-2–recruited proMMP-2 at the cell surface [41]. Its strategic localization at the plasma membrane also increases the likelihood of interaction with other membrane-bound receptors and cofactors, enabling MMP-14 to integrate proteolytic activity with signaling functions at sites of cell–matrix contact. MMP-14 also exerts broader biological effects that extend beyond extracellular matrix remodeling. Through both proteolytic and non-proteolytic mechanisms, it influences cellular apoptosis, immune responses, and tissue homeostasis by modulating cell survival, inflammatory signaling, and stromal–immune interactions. Consequently, aberrant MMP-14 activity contributes to the progression of diverse pathological conditions, and elucidating the molecular mechanisms governing MMP-14 function may reveal novel therapeutic opportunities across multiple disease contexts [17]. Collectively, these biochemical and functional features position MMP-14 as a key regulator of tissue remodeling, morphogenesis, and angiogenesis. Dysregulation of MMP-14 activity has been implicated in a wide range of pathological conditions, including atherosclerosis, aneurysm formation, cancer progression, neuroinflammation, and macular degeneration [18,42,43,44].

#### Structural Basis of Inhibitor Selectivity

Matrix metalloproteinases share a highly conserved three-dimensional catalytic domain architecture that underlies their proteolytic activity and shapes inhibitor selectivity. The catalytic domain adopts a compact globular fold composed of a five-stranded β-sheet flanked by three α-helices, stabilized by structural calcium ions. The active site contains a catalytic Zn^2+^ ion coordinated by three histidine residues within the conserved HEXGHXXGXXH motif, with a catalytic glutamate serving as a general base during peptide bond hydrolysis. Substrate recognition is largely governed by the S1′ specificity pocket, a hydrophobic cavity adjacent to the catalytic zinc that accommodates the P1′ residue of substrates and inhibitors. Although the overall fold is conserved across the MMP family, variations in the depth, width, and flexibility of the S1′ pocket—primarily dictated by loop regions surrounding the active site—create important determinants of substrate preference and inhibitor selectivity. For example, MMP-13 possesses a deep and flexible S1′ tunnel due to conformational mobility of the Ω-loop, whereas membrane-type MMPs such as MMP-14 display a medium-depth pocket shaped by MT-specific loop features. Subtle differences in pocket-lining residues and loop dynamics, rather than gross architectural changes, therefore, represent the principal structural basis for isoform-selective inhibitor design. These conserved yet tunable structural elements provide both opportunities and challenges for developing selective therapeutic inhibitors, particularly for closely related family members such as the membrane-type MMPs.

### 3.2. Subcellular Localization and Pericellular Proteolysis

A defining feature of MMP-14 is its membrane anchoring, which enables precise pericellular proteolysis and distinguishes MT-MMPs from soluble MMPs [45]. MMP-14 localizes to dynamic membrane protrusions that are structurally essential for directed cell migration and ECM degradation, including lamellipodia, filopodia, podosomes, and invadopodia, the latter classically described in tumor cells [46,47,48,49]. At lamellipodia, MMP-14 forms a complex with CD44 via its hemopexin domain, linking the enzyme to the actin cytoskeleton and stabilizing its position at the leading edge. This anchoring allows MMP-14 to degrade the adjacent ECM while maintaining spatial orientation during cell movement [50]. In invadopodia, the major sites of localized ECM degradation, MMP-14 is the principal protease responsible for generating focal proteolytic zones that promote cellular invasion [48]. An 8-amino-acid MT-Loop within its catalytic domain is crucial for mediating interactions with adhesion complexes and for proper localization to these structures [51]. In addition to its surface distribution, MMP-14 has been detected in intracellular compartments, including the cytoplasm, nucleus, Golgi apparatus, and caveolae [52]. This broad subcellular localization suggests that MMP-14 participates not only in extracellular matrix turnover but also in intracellular signaling, metabolic regulation, and trafficking processes.

### 3.3. Regulation of Expression, Activation, and Trafficking

MMP-14 activity is regulated at multiple levels, including transcriptional regulation, proteolytic activation and intracellular trafficking, cell-surface regulation, and inhibition by TIMPs [53,54].

#### 3.3.1. Transcriptional Regulation

Most matrix metalloproteinases are transcriptionally regulated by promoters containing a canonical TATA box located approximately 30 bp upstream of the transcription start site, typically in conjunction with an AP-1 binding site and, in many cases, a nearby PEA3 element that cooperates with AP-1 to drive gene expression [55]. In contrast, MMP-14 belongs to a distinct subset of MMPs, together with MMP-2, that lack a TATA box and initiate transcription from multiple start sites. Instead, *MMP-14* transcription is primarily dependent on a GC-rich Sp1 binding site, deletion of which abolishes approximately 90% of promoter activity [54]. This Sp1 site partially overlaps with an Early Growth Response-1 (Egr-1) binding element, allowing Egr-1 to compete with Sp1 and function as an inducible transcriptional activator of *MMP-14* in a cell–type–specific manner, particularly in endothelial cells [56].

In addition to Sp1 and Egr-1, multiple transcription factors modulate *MMP-14* expression, including AP-4, NF-κB, Nkx-2 [56,57], ETS family members [58], and p53 [59]. *MMP-14* expression is further regulated by epigenetic and post-transcriptional mechanisms, such as DNA methylation [60] and microRNA-mediated control [53], underscoring the complexity of its transcriptional regulation across different cellular contexts.

Although most transcriptional regulators act as activators of the *MMP-14* promoter, several factors function as negative regulators. Both p53 [59] and PROX1 [61] repress *MMP-14* transcription in specific cell types. In glioblastoma cells, suppression of NF-κB signaling by the histone acetyltransferase Tip60 leads to reduced *MMP-14* transcription [57]. Conversely, in breast and ovarian cancer cells, Rab coupling protein activates a β1-integrin/EGFR/Src/STAT3 signaling cascade that enhances *MMP-14* expression through NF-κB activation [62]. In metastatic melanoma, the VPS35/retromer complex regulates MMP-14 transcription via activation of the inflammatory IL-6/STAT3 pathway, with VPS35 expression positively correlating with MMP-14 levels [63]. Additionally, TGF-β1 induces *MMP-14* transcription through Smad-dependent signaling, with ERK1/2 and p38 MAPK pathways modulating promoter activity and protein expression in oral squamous cell carcinoma models [64].

Hypoxia-inducible factor-1 (HIF-1) directly upregulates *MMP-14* transcription by binding to hypoxia-responsive elements within the *MMP-14* promoter, thereby linking hypoxic microenvironments to enhanced MMP-14-mediated extracellular matrix remodeling and angiogenesis [65].

*MMP-14* expression is further regulated by epigenetic mechanisms. Hypermethylation of CpG islands within the *MMP-14* promoter, together with histone H3 lysine-27 trimethylation (H3K27me3), leads to transcriptional silencing of *MMP-14*, whereas promoter hypomethylation and reduced H3K27me3 are associated with transcriptional activation in cancer cells [60]. In addition, several microRNAs—including miR-484 [66], miR-485-5p [67], and miR-142-5p [68], negatively regulate *MMP-14* expression by directly targeting its mRNA. In cervical cancer cells, DNMT1-mediated hypermethylation of the miR-484 promoter suppresses miR-484 expression, relieving post-transcriptional repression of *MMP-14* and contributing to its upregulation [66].

#### 3.3.2. Proteolytic Activation and Intracellular Trafficking

MMP-14 is synthesized as an inactive zymogen and undergoes proteolytic activation within the trans-Golgi network, where its N-terminal propeptide is cleaved by furin-like proprotein convertases to generate the mature, catalytically active enzyme [69]. Following activation, MMP-14 is packaged into vesicles and trafficked to the plasma membrane through tightly regulated intracellular transport mechanisms. The delivery of MMP-14–containing vesicles is coordinated by members of the Rab family of small GTPases, which regulate vesicle budding, transport, and targeting [70]. Microtubule-based kinesin motor proteins, including KIF5B and the KIF3A/KIF3B complex, further facilitate directional transport of MMP-14 toward the cell periphery [71]. In addition, KIF13A together with KIF3A has also been implicated in targeted transport of MMP-14–containing vesicles to focal adhesions [71,72]. The Golgi reassembly stacking protein GRASP55 plays a crucial role in post-Golgi trafficking by regulating vesicle maturation and efficient delivery of MMP-14 to the plasma membrane [73]. Together, proteolytic activation and coordinated intracellular trafficking ensure that active MMP-14 is precisely delivered to the cell surface, where it can exert spatially restricted pericellular proteolysis critical for extracellular matrix remodeling, cell migration, and angiogenic processes.

#### 3.3.3. Cell-Surface Regulation

At the plasma membrane, MMP-14 activity and surface availability are tightly regulated through interactions with other membrane-associated proteases, integrins, and scaffolding proteins. One well-characterized regulatory axis involves ADAM12 and αvβ3 integrin. Albrechtsen et al. [74] demonstrated that ADAM12 controls both the surface expression and proteolytic activity of MMP-14 by forming a ternary complex with αvβ3 integrin in human breast cancer cells [74]. Disruption of ADAM12 function using blocking antibodies significantly impaired MMP-14-mediated gelatin degradation, suggesting that ADAM12 positively regulates MMP-14 activity at the cell surface. In addition, MMP-14 functions as a sheddase for the membrane-tethered metalloprotease meprin β, while meprin β can reciprocally shed MMP-14. MMP-14, ADAM10, and ADAM17 all cleave meprin β at a shared site between Pro602 and Ser603, located N-terminal to the EGF-like domain [75]. In contrast, MMP-14 can also act as a negative regulator of other ADAM family members. In osteoblasts, MMP-14 forms a complex with fibroblast growth factor receptor 2 (FGFR2) and ADAM9, leading to inactivation of ADAM9 and attenuation of ADAM9-mediated FGFR2 shedding at the cell surface [76]. These findings underscore the context-dependent regulatory crosstalk between MMP-14 and other membrane-associated metalloproteases.

Integrins represent another major class of regulators that control MMP-14 localization and function at adhesion sites. MMP-14 is recruited to integrin-rich focal adhesions, and disruption of its MT-loop impairs surface activity by preventing efficient localization to β1-integrin–containing adhesion complexes [77]. In addition to being regulated by integrins, MMP-14 can proteolytically process the pro-forms of multiple α-integrins (αv, α3, α5, α6, α7, α8, and α9), thus modulating integrin maturation and adhesive function [51]. In invasive tumor cells, β1-integrin activation triggers Src/EGFR signaling that promotes MMP-14 phosphorylation, internalization, and recycling to invadopodia, facilitating localized extracellular matrix degradation [78]. Conversely, inhibition of β1-integrin increases its association with MMP-14 at the cell surface, sequestering both proteins and impairing their internalization and recycling, which in turn reduces pericellular proteolysis [78]. Additional membrane-associated proteins further refine MMP-14 surface distribution. CD44 has been shown to regulate MMP-14 positioning through a direct interaction between the CD44H isoform and the hemopexin (PEX) domain of MMP-14. The cytoplasmic tail of CD44H links this complex to the actin cytoskeleton, directing MMP-14 to lamellipodia and promoting directional cell migration [50]. This PEX-dependent interaction is also required for efficient CD44 shedding by MMP-14 [50]. Tetraspanins further influence MMP-14 trafficking and membrane organization. Several tetraspanin family members—including CD9, CD37, CD53, CD63, CD81, and CD82—associate with MMP-14 via its hemopexin domain within the endoplasmic reticulum, thereby regulating its surface delivery, spatial organization, and proteolytic activity [79]. Finally, caveolin-1, a core structural component of caveolae, negatively regulates MMP-14 by promoting its internalization from the plasma membrane, thus limiting sustained surface proteolysis [80].

#### 3.3.4. Inhibition by TIMPs

MMP-14 is post-translationally regulated by members of the tissue inhibitor of metalloproteinases (TIMP) family, which exert differential and context-dependent effects on its activity. Among these, TIMP-2 is the primary endogenous regulator of MMP-14, exhibiting a unique dual function as both an activator and an inhibitor. At low concentrations, TIMP-2 facilitates pro-MMP-2 activation by forming a ternary complex with MMP-14 and pro-MMP-2, positioning the zymogen for efficient cleavage by an adjacent MMP-14 molecule [81]. At higher concentrations, however, TIMP-2 directly inhibits MMP-14 catalytic activity. This functional duality arises from distinct binding interactions mediated by the N-terminal inhibitory domain and the C-terminal domain of TIMP-2 with the hemopexin (PEX) domain of MMP-14 [82,83]. In contrast, TIMP-1 exhibits relatively low affinity for membrane-type MMPs and shows little to no measurable inhibition of MMP-14 at physiologically relevant concentrations, likely due to an unfavorable conformational entropy penalty upon binding [84]. TIMP-3, a matrix-bound TIMP, can inhibit MMP-14 and has been implicated in the regulation of pericellular proteolysis [85]. In addition to direct inhibition, TIMP-3 promotes clearance of MMP-14 from the cell surface by facilitating its association with low-density lipoprotein receptor–related protein-1 (LRP-1), thereby enhancing MMP-14 endocytosis and limiting sustained surface activity [86]. TIMP-4 is a potent inhibitor of MMP-14, effectively blocking the MMP-14–dependent activation of pro-MMP-2 [87]. Unlike TIMP-2, TIMP-4 does not support pro-MMP-2 activation and instead competitively suppresses this process when both inhibitors are present [88]. Collectively, these differential TIMP interactions provide a finely tuned regulatory network that controls MMP-14 activity, localization, and downstream proteolytic signaling in a concentration- and context-dependent manner.

### 3.4. Non-Proteolytic Functions

Emerging evidence indicates that MMP-14 exerts important non-proteolytic functions that extend beyond extracellular matrix degradation. These activities are mediated primarily through its cytoplasmic tail and its capacity to interact with intracellular signaling partners, positioning MMP-14 as a multifunctional regulator of cellular behavior [52].

#### 3.4.1. Macrophage Motility and Metabolism

The cytoplasmic tail of MMP-14 is essential for macrophage migration and metabolic regulation. Macrophages lacking MMP-14 exhibit impaired motility that is independent of its catalytic activity, indicating a non-enzymatic structural or scaffolding role for the protein [65,89]. These findings demonstrate that MMP-14 contributes to immune cell dynamics through mechanisms distinct from its proteolytic function.

#### 3.4.2. Regulation of HIF-1α Signaling

Hypoxia-inducible factors (HIFs) are transcription factors whose stabilization and activity are regulated by oxygen availability and which control gene programs involved in glucose metabolism, angiogenesis, migration, and invasion [65]. At the Golgi apparatus, the cytoplasmic tail of MMP-14 interacts with factor inhibiting HIF-1 (FIH-1), enabling munc-18-1-interacting protein 3 (Mint3) to suppress FIH-1 activity. This interaction promotes HIF-1α activation and establishes a reciprocal regulatory loop in which HIF-1α, in turn, enhances *MMP-14* transcription under hypoxic conditions [90]. This pathway is specific to HIF-1α regulation and provides a clear example of protease-independent, cytoplasmic-tail–dependent control of transcriptional programs.

#### 3.4.3. Protease-Independent Gene Regulation

Protease-independent roles of MMP-14 have also been linked to changes in gene expression [52]. Although nuclear localization has been reported in some settings, mechanistic evidence in macrophages supports a signaling-mediated effect on transcriptional programs. In this context, MMP-14 modulates the expression of more than 100 genes, a substantial proportion of which are involved in immune regulation, at least in part via activation of the PI3Kδ/Akt/GSK3β signaling axis in macrophages [91]. These findings further expand the functional repertoire of MMP-14 beyond extracellular proteolysis, highlighting its role as a regulator of intracellular signaling and transcriptional programs.

## 4. MMP-14-Mediated Regulation of Angiogenic Signaling

### 4.1. MMP-14 Regulation of VEGF/VEGFR Signaling in Angiogenesis

Vascular endothelial growth factor (VEGF) signaling is a central driver of vascular development, endothelial survival, and pathological angiogenesis, including CoNV. VEGF ligands—particularly VEGF-A—are produced by stromal, endothelial, and inflammatory cells in response to hypoxia and inflammatory cytokines. VEGFs signal through three type III receptor tyrosine kinases: VEGFR1 (Flt-1), VEGFR2 (KDR/Flk-1), and VEGFR3 (Flt-4). Ligand binding induces receptor homo- or heterodimerization and activates distinct downstream signaling programs [92]. VEGFR2 is the principal mediator of angiogenic responses. Engagement of VEGF-A with VEGFR2 drives endothelial proliferation, migration, survival, cytoskeletal remodeling, and vascular permeability through canonical PLCγ–MAPK, PI3K/Akt, Src–FAK, and p38/SAPK pathways [93]. Accordingly, VEGFR2 signaling is indispensable for embryonic vasculogenesis and adult sprouting angiogenesis. In contrast, VEGFR1 primarily serves a modulatory role. Although it binds VEGF-A with high affinity, VEGFR1 exhibits relatively weak tyrosine kinase activity and mainly regulates angiogenic patterning by shaping VEGFR2 signaling rather than acting as a dominant mitogenic receptor. Its soluble isoform, sVEGFR1 (sFlt-1), functions as a decoy receptor that sequesters VEGF-A and limits VEGFR2 activation. In addition, VEGFR1 contributes to inflammatory angiogenesis by mediating monocyte recruitment [94]. Collectively, VEGF/VEGFR signaling establishes the endothelial activation program that governs vessel sprouting, branching, and stabilization. Because VEGFR2 drives pro-angiogenic signaling while VEGFR1 regulates ligand availability and signaling balance, perturbations affecting either receptor can profoundly alter angiogenic outcomes—particularly in pathological contexts such as CoNV. Within this tightly regulated system, proteolytic enzymes that modify receptor abundance or ligand accessibility can exert powerful control over angiogenesis. MMP-14 is one such regulator, acting through context-dependent cleavage of VEGFR1 and extracellular vesicle–mediated mechanisms [93,95].

#### MMP-14-Mediated Cleavage of VEGFR1

**Figure 2:**
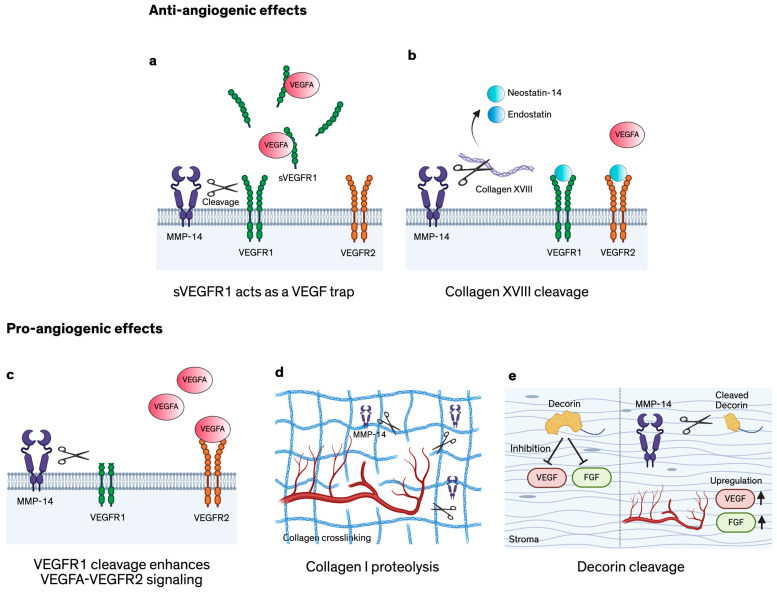
Pro- and anti-angiogenic mechanisms mediated by membrane-associated MMP-14 at the cell surface. Top panels (anti-angiogenic): (**a**) MMP-14 cleavage of VEGFR1 generates soluble VEGFR1, which sequesters VEGF-A and acts as a VEGF trap [96]; (**b**) MMP-14 cleavage of collagen XVIII releases anti-angiogenic fragments (endostatin and neostatin-14) [101]. Bottom panels (pro-angiogenic): (**c**) MMP-14 cleavage reduces membrane VEGFR1 decoy function, shifting VEGF-A signaling toward VEGFR2 [97]; (**d**) MMP-14–mediated collagen I remodeling modulates stromal matrix organization [5]; (**e**) MMP-14 cleavage of decorin decreases growth-factor sequestration, increasing VEGF and FGF bioavailability in the stroma [102].

In corneal experimental models, the catalytic domain of MMP-14 cleaves VEGFR1 within its extracellular region at two sites, generating three discrete fragments [96] (Figure 2). The pro- and anti-angiogenic outcomes described below were reported in different experimental settings, and they will predominate in corresponding clinical settings. In vitro assays using the catalytic domain of MMP-14 showed that MMP-14 cleaves VEGFR1 and cuts off the N-terminal extracellular portion of the receptor, releasing it as a soluble fragment that can still bind VEGF-A165. This fragment suppressed VEGF-A165-induced endothelial proliferation and p-ERK activation, consistent with a ‘VEGF-trap’ effect [96]. By contrast, another experimental setting analyzed MMP-14 delivered via exosomes produced by corneal fibroblasts. In this study, exosomes containing MMP-14 promoted VEGFR1 cleavage in human umbilical vein endothelial cells (HUVECs) without inducing VEGFR2 cleavage, and exposure was associated with increased endothelial migration and VEGFA-induced proliferation [97]. Because VEGFR1 can act as a decoy receptor for VEGFR2, the authors suggest that VEGFR1 cleavage may facilitate greater VEGFR2 activation upon VEGFA binding [97]. Given the central role of VEGFA in injury-induced CoNV in murine models [98], the authors further propose that exosomes may serve as a natural delivery vehicle for transferring active MMP-14 to endothelial cells, providing a mechanism of fibroblast-to-endothelial communication in VEGFA-driven corneal neovascularization [97]. Thus, the predominant hypothesis is that the net clinical effect of MMP-14–mediated cleavage of VEGFR1 depends on which scenario dominates: accumulation of soluble VEGF-binding VEGFR1 fragments that sequester VEGF (for example, in early stages of corneal wound healing [99]), or preferential VEGFR1 cleavage near endothelial cells that enhance VEGFR2 activation (for example, in overactivation of MMP-14 on the cell surface VEGFR1 in the setting of CoNV associated with prolonged injury [100]).

### 4.2. MMP-14 Cleavage of Collagen XVIII Inducing Apoptosis of Endothelial Cells

MMP-14 also modulates angiogenesis by proteolytically processing extracellular matrix–derived anti-angiogenic factors. Collagen XVIII is cleaved by MMP-14 to generate endostatin and neostatin-14 [101]. Endostatin is a 22 kDa peptide derived from the C-terminal region of the collagen XVIII α1 chain and is a well-established endogenous inhibitor of angiogenesis. It binds VEGFR2, attenuating VEGF-A–induced receptor phosphorylation and downstream signaling and also interferes with VEGF-A binding to VEGFR1 [103,104]. Through collagen XVIII cleavage, MMP-14 generates endostatin-like fragments that disrupt key endothelial processes, including migration and proliferation. Both endostatin and neostatin-14 inhibit basic fibroblast growth factor (bFGF)–induced corneal neovascularization and promote endothelial cell apoptosis [101]. These anti-angiogenic fragments contribute to maintenance of corneal avascularity—often termed “corneal angiogenic privilege”—highlighting a counterbalancing role for MMP-14 in restraining excessive neovascularization [8]. Thus, MMP-14 participates in both pro- and anti-angiogenic pathways, reinforcing its context-dependent regulatory role in corneal angiogenesis.

### 4.3. MMP-14–Mediated Cleavage of EphA2 and Loss of Vascular Quiescence

MMP-14 has also been shown to regulate angiogenesis by proteolytically processing EphA2, a receptor tyrosine kinase that normally contributes to vascular stability and quiescence. In several cellular contexts, EphA2 functions as a tumor suppressor and maintains endothelial organization [105]. MMP-14 cleaves EphA2 within its extracellular fibronectin type-III domain, converting it into a truncated, pro-oncogenic form [106,107]. This cleavage induces intracellular EphA2 relocalization and activates signaling pathways that enhance cell migration, invasion, and anchorage-independent growth.

In vascular endothelial cells, full-length EphA2 promotes vessel stability, whereas its truncated form increases endothelial motility and disrupts quiescence, which creates conditions favorable for angiogenesis. In the cornea, where angiogenic privilege is actively maintained, elevated MMP-14 expression and subsequent EphA2 cleavage may undermine vascular stability, promote endothelial activation, and trigger CoNV [107]. These findings further underscore the capacity of MMP-14 to modulate angiogenesis by reprogramming receptor signaling at the endothelial cell surface.

## 5. Roles of MMP-14 in the Cornea

**Figure 3:**
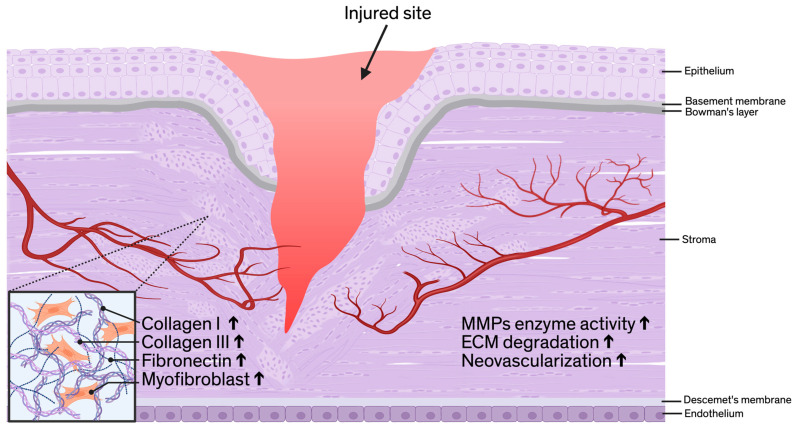
Injury-induced stromal remodeling and corneal neovascularization. Schematic representation of the corneal injury microenvironment illustrating epithelial disruption, stromal remodeling, and ingrowth of new blood vessels. The injured stroma is characterized by increased deposition of collagens I and III and fibronectin, myofibroblast accumulation, enhanced MMP enzymatic activity, and extracellular matrix (ECM) degradation, with associated loss of stromal organization.

MMP-14 is a critical modulator of corneal physiology and pathology, particularly in dynamic processes such as ECM remodeling, wound healing, neovascularization, and lymphangiogenesis [31,82,96,100]. Clinically, CoNV reduces corneal transparency and can impair vision, and it commonly arises in settings of infection, inflammation, hypoxia, or injury [10]. More broadly, these conditions can disrupt ECM homeostasis, promote scarring, and trigger abnormal growth of both blood and lymphatic vessels in the cornea [10,99,108]. Therefore, clarifying how MMP-14 regulates pericellular ECM remodeling and growth factor/receptor signaling in the cornea is directly relevant to wound healing outcomes, stromal fibrosis, and pathological vessel growth. Figure 3 schematically depicts the injury site microenvironment, highlighting upregulation of stromal matrix components, myofibroblast accumulation, increased MMP activity, and associated pathological neovascularization.

As a membrane-anchored metalloproteinase, MMP-14 exerts pericellular proteolytic activity, most notably the degradation of type I collagen and activation of pro-MMP-2 [4], facilitating tissue remodeling during homeostasis and after injury. Its expression is upregulated in response to corneal damage, especially in stromal keratocytes, and persists throughout the wound healing process [99]. Besides its role in ECM turnover, MMP-14 also regulates signaling cascades by modulating growth factors and receptors, including VEGFA and VEGFR1 [3,100]. MMP-14 has a pro-angiogenic function in the cornea by potentiating bFGF-induced corneal neovascularization and promoting the secretion of VEGFA [100].

MMP-14 influences the VEGF, bFGF, platelet-derived growth factor (PDGF), hepatocyte growth factor (HGF), chemokines, Wnt/β-catenin, TGF, EGF, PGE2, thrombin, Notch, integrins, TLR, PI3k/Akt, Src, RhoA/ROCK, and ERK signal transduction pathways, which regulate angiogenesis and ECM remodeling [82,109]. Depending on the local context, MMP-14 may also suppress neovascularization by generating soluble receptor fragments that act as a “VEGF trap” and limit VEGF-A availability [96]. Moreover, MMP-14 can be transferred between cells via exosomes secreted by corneal fibroblasts, expanding its functional reach to cells that do not express it naturally [96]. The following sections provide a detailed examination of the specific roles of MMP-14 in corneal wound healing, corneal angiogenesis, and lymphatic vessel formation.

### 5.1. The Role of MMP-14 in Corneal Injury and Wound Healing

MMP-14 is expressed both in normal and wounded corneas [99]. In situ hybridization localized MMP-14 mRNA predominantly to stromal keratocytes, with only rare staining in basal epithelial cells. Following excimer laser keratectomy in rats, elevated MMP-14 expression was observed at 1, 3, and 14 days in keratocytes after wounding [99]. One of its main functions is the activation of pro-MMP-2 at the stromal cell surface [4], which plays a role in the prolonged process of collagen remodeling and supports tissue regeneration [110] in the corneal stroma. Since MMP-2 displays a similar expression pattern (upregulated in superficial stromal keratocytes at days 3 and 7 after injury and weakly expressed in the epithelium) [99], MMP-14 may activate pro-MMP-2 locally, on the surface of stromal cells. Thus, MMP-14 likely contributes to the regulation of ECM remodeling by maintaining the balance between matrix degradation and synthesis, an essential aspect for effective tissue turnover and repair during corneal wound healing [99].

In addition to its role in pro-MMP-2 activation, MMP-14 contributes directly to the remodeling of the fibrotic matrix through the degradation of structural ECM components [110]. In a model of corneal injury, the overexpression of MMP-14 resulted in the reduction in stromal scarring and improved corneal transparency, suggesting a protective effect against excessive collagen accumulation [111]. Also, MMP-14 overexpression attenuated the expression of major genes involved in corneal scarring, particularly type III collagen and α-smooth muscle actin [111]. Galiacy et al. [111] hypothesized that this antifibrotic effect could be attributed to a feedback mechanism in which MMP-14-mediated remodeling of the ECM decreases stromal stiffness by degrading type III collagen. This resulting reduction in ECM stiffness may inhibit myofibroblasts, which could explain the decrease in a-SMA and type III collagen mRNA [111].

### 5.2. MMP-14 Angiogenic Effects Are Context-Dependent

MMP-14 plays a multifaceted role in corneal angiogenesis, a complex but ordered and step-dependent process [10]. As potentially the most critical MMP regulating VEC’s functions [3], MMP-14 functions as a crucial mediator in maintaining the angiogenic equilibrium of the cornea, with the capacity to either promote or suppress neovascularization, depending on the context. The pro-angiogenic mechanisms of MMP-14 include, but are not limited to: (1) cleavage of ECM molecules [4], (2) upregulation of VEGF [3], (3) interaction with cell surface mediators such as CD44 [3], (4) cleavage of anti-angiogenic molecules such as decorin [102], (5) activation of pro-MMP-2, contributing to matrix degradation and facilitating vessel sprouting [96], (6) regulation of VEGF receptor signaling [100]. The anti-angiogenic effects associated with MMP-14 are primarily represented by its capacity to cleave VEGFR1, leading to a VEGF-trap mechanism [96], and by processing collagen XVIII to generate neostatin-14, a fragment that inhibits endothelial proliferation and migration [101].

In addition to the aforementioned functions, MMP-14 actively remodels the stromal environment and enables VECs invasion and vessel formation [5]. Chun et al. [5] demonstrated that the cleavage of type I collagen, mediated by MMP-14, facilitates VECs migration, capillary sprouting, and tubulogenic organization in an ex vivo three-dimensional model. This reshaping of the collagen structure by MMP-14 is conducted in a controlled way, generating spatial guidance that directs new vessel growth. Remodeling of the ECM is a key early step in neovascularization. Stratman et al. [112] further supported these findings by demonstrating, in a three-dimensional model, that MMP-14 is critical for generating vascular guidance structures that facilitate endothelial lumen formation, direct cell migration, and support tube remodeling, allowing endothelial cells to organize into stable vessel walls [112]. These structures arise through the focal degradation of collagen type I by catalytically active MMP-14 and activation of pro-MMP-2. Together, these studies demonstrate that MMP-14 guides the early stages of angiogenesis by remodeling type I collagen to guide VECs migration, lumen formation and vessel stabilization.

MMP-14 initiates the pro-angiogenic cascade at the molecular signaling level, by upregulating intracellular VEGF-A expression [113], a mediator that strongly contributes to CoNV [114]. Han et al. [113] showed that MMP-14 is required for bFGF-induced upregulation of VEGF-A expression in a model of cultured corneal fibroblasts. In MMP-14 knockout fibroblasts, bFGF-induced VEGF-A mRNA expression was significantly reduced compared with knock-in fibroblasts with overexpression of MMP-14. The authors reported that MMP-14 modulates Ras and ERK, key molecules involved in the interposition of the bFGF-induced VEGF-A expression cascade in corneal fibroblasts [113].

MMP-14 promotes a pro-angiogenic stromal environment in part by degrading endogenous anti-angiogenic factors. One of its key substrates is decorin, a leucine-rich small proteoglycan abundantly expressed in the normal corneal stroma [102,115]. Decorin is a well-established inhibitor of angiogenesis: it suppresses VEGF- and bFGF-driven neovascularization [116], impedes endothelial cell migration, and prevents vascular tube formation [117]. Mimura et al. [102] demonstrated that MMP-14 directly cleaves decorin in vitro in a time- and concentration-dependent manner, generating fragments that lack the parent molecule’s anti-angiogenic activity. Functionally, MMP-14 reversed the inhibitory effects of decorin on endothelial cell proliferation and migration, thereby facilitating angiogenic responses. In vivo, corneas with experimentally increased MMP-14 activity exhibited reduced decorin expression in regions undergoing neovascularization, further supporting the idea that MMP-14 shifts the stromal milieu toward an angiogenic state by removing decorin’s inhibitory influence [102].

In addition to its pro-angiogenic functions, evidence from MMP-14-deficient mice reveals that MMP-14 is indispensable for the initiation of angiogenesis in vivo. Zhou et al. [118] demonstrated that mice lacking MMP-14 exhibit a complete absence of FGF-2–induced corneal neovascularization in the micropocket assay, despite normal VEGF and VEGFR2 (Flk-1) expression in adjacent tissues. Corneal pellets containing FGF-2 triggered robust vascular growth in wild-type mice, yet null mice showed no angiogenic response, indicating a strict requirement for MMP-14 in the earliest steps of vessel sprouting [118]. This failure of neovascularization paralleled defects in other vascularization processes—such as impaired blood-vessel invasion into hypertrophic cartilage—implying that MMP-14 is essential for basement-membrane degradation and endothelial invasion during angiogenic initiation. These findings highlight that loss of MMP-14 activity can confer a strong anti-angiogenic phenotype, underscoring its dual capacity to both promote and, when absent, suppress neovascularization depending on the context.

Beyond initiating vessel sprouting, MMP-14 is essential for the physiological stability and maturation of newly formed vessels. A central element of new vessel maturation is mural cell investment (pericytes and vascular smooth muscle cells), which depends on PDGF-B/PDGFRβ signaling [119] and seems to be regulated by MMP-14 [120]. Lehti et al. [120] demonstrated that MMP-14 is associated with PDGFRß in vascular smooth muscle cells and regulates PDGF-B signal transduction cascades. Consequently, MMP-14-deficient tissues displayed attenuated PDGF-B-dependent cellular function and therefore exhibited severe defects in pericyte association, with regressing and dilated capillary structures [120].

MMP-14 also modulates angiogenesis through interactions with other proteins beyond the classical ECM substrates. One example is endoglin (CD105), a membrane receptor highly expressed on proliferating VECs and with a crucial role in angiogenesis [121]. Hawinkels et al. [2] demonstrated that MMP-14 is a key endoglin-shedding protease, and the coexpression of endoglin with MMP-14 on the cell membrane resulted in the cleavage of endoglin. In addition, knockdown of MMP-14 reduced soluble endoglin levels in vitro, which demonstrated anti-angiogenic properties [2]. Furthermore, MMP-14 has been shown to cooperate with sphingosine 1-phosphate (S1P). This interaction promotes VECs cell migration and capillary morphogenesis, further diversifying the repertoire of MMP-14 in angiogenesis [122].

Azar et al. [123] reported that corneal epithelial MMP-14 exerts anti-angiogenic effects independent of its proteolytic activity [123]. In vitro, conditioned media from MMP-14–deficient epithelial cells enhanced calf pulmonary arterial endothelial cell proliferation and migration compared with media from wild-type cells, whereas media from MMP-14 knock-in cells reversed these effects. Notably, overexpression of a catalytically inactive MMP-14 mutant (MMP-14-E240A) similarly suppressed endothelial proliferation and migration, indicating that this anti-angiogenic function does not require enzymatic activity. These findings suggest that non-catalytic domains of MMP-14, including its transmembrane and cytoplasmic regions, contribute to epithelial-mediated angiogenic regulation in this context. Table 1 summarizes the experimental evidence for the MMP-14–related angiogenic mechanisms.

### 5.3. Role of MMP-14 in Corneal Lymphangiogenesis

Lymphangiogenesis is the process by which new lymphatic vessels form, playing a critical role in corneal wound healing. MMP-14 expression increases after corneal injury and appears to modulate this process. Evidence from Wong et al. [108] indicates that MMP-14 can act as a negative regulator of corneal lymphangiogenesis [108]. The study showed that MMP-14 reduces VEGF-C production by macrophages and cleaves lymphatic vessel endothelial hyaluronan receptor-1 (LYVE-1) from the surface of lymphatic endothelial cells, thereby limiting lymphatic vessel growth and stability. In MMP-14-deficient mice, the absence of this regulatory control resulted in enhanced lymphangiogenesis under inflammatory conditions [108].

However, other mechanisms may also contribute to how MMP-14 influences the promotion or inhibition of lymphangiogenesis. Ingvarsen et al. [124] developed a monoclonal antibody that selectively inhibits the MMP-14-mediated activation of pro-MMP-2 without affecting its general proteolytic or collagenolytic activity [124]. Using this antibody, the authors demonstrated that blocking pro-MMP-2 activation inhibited the outgrowth of cultured lymphatic endothelial cells in a collagen matrix in vitro and lymphatic vessel sprouting in an ex vivo model. These findings identified MMP-14-dependent pro-MMP-2 activation as an important step in lymphatic endothelial cell sprouting [124]. In addition, Alderfer et al. [125] showed that MMP-14 is required for the formation of lymphatic structures in lymphatic endothelial cells, with its expression regulated by matrix stiffness and VEGF-C [125].

Together, these studies demonstrate that MMP-14 may influence lymphangiogenesis through multiple mechanisms, with its function depending on the environmental context.

## 6. Treatment of Corneal Neovascularization

### 6.1. Current Therapies for CoNV

Corneal neovascularization arises when tissue damage or inflammation disrupts the cornea’s angiogenic privilege and induces the release of pro-angiogenic factors, most prominently VEGF. Current therapies primarily target inflammation or VEGF-driven signaling, although clinical efficacy remains variable [126].

Topical dexamethasone, one of the most commonly used agents, suppresses inflammatory pathways that drive corneal angiogenesis [127]. However, its therapeutic effect is often incomplete, in part due to limited corneal penetration and poor water solubility [128]. Chronic use carries well-known risks, including corneal thinning, ocular hypertension, cataracts, and activation of herpes simplex keratitis [126]. Other corticosteroids, such as loteprednol etabonate, betamethasone acetate, fluorometholone, and prednisolone, are suggested as alternatives depending on desired potency and safety profiles [127].

Nonsteroidal anti-inflammatory drugs (NSAIDs), including diclofenac and ketorolac, inhibit prostaglandin synthesis and may reduce angiogenic responses [127]. However, their clinical benefit in CoNV is limited. Prolonged use may impair epithelial wound healing, highlighting the need for caution in patients with active corneal surface defects [129].

Topical immunomodulatory agents offer steroid-sparing options for chronic or immune-mediated keratitis [127]. Cyclosporine A and tacrolimus reduce neovascularization by inhibiting T-cell activation and suppressing the production of inflammatory cytokines. These drugs are particularly useful in cases of recurrent inflammation or when long-term corticosteroid exposure is undesirable [127,130,131].

Beyond angiogenic signaling, MMP-14 also links inflammation and matrix remodeling in the cornea, processes that commonly coexist in pathological neovascularization [127]. Pro-inflammatory mediators implicated in CoNV, including IL-6/STAT3- and TGF-β–related programs, can modulate MMP-14 expression and activity [82]. Conversely, MMP-14 can influence inflammatory cell behavior by supporting pericellular matrix remodeling and macrophage motility through proteolytic and cytoplasmic-tail–dependent mechanisms [91,132]. Therefore, anti-inflammatory therapies may reduce MMP-14–associated remodeling indirectly by suppressing upstream inflammatory drivers, even though they do not inhibit MMP-14 directly.

Anti-VEGF biologics have emerged as an off-label therapeutic option for CoNV. Bevacizumab (anti-VEGF-A) has demonstrated regression of established corneal vessels in clinical and experimental studies [133]. In a pilot study, bevacizumab significantly reduced CoNV in most treated eyes within one month [134]. Other VEGF inhibitors, such as ranibizumab and aflibercept, have also shown efficacy in experimental and clinical settings [135,136]. Despite these benefits, anti-VEGF therapy requires repeated dosing and may cause adverse effects, including corneal thinning, epithelial erosion, and, in some cases, diminished responsiveness over time [134]. These limitations illustrate the need for additional therapeutic strategies, particularly those that can modulate upstream proteolytic pathways, normalize stromal remodeling, or target non-VEGF angiogenic drivers.

Small molecule inhibitors directed against MMPs represent one such approach. Because MMP-14 and related MMPs regulate key steps in endothelial invasion, ECM degradation, and VEGF/VEGFR signaling, selective inhibition of these enzymes offers a promising strategy to suppress CoNV at an earlier and more mechanistic level than current anti-VEGF or anti-inflammatory therapies. Several MMP-targeting compounds are now being evaluated for their potential to block pathological corneal angiogenesis [126].

From a translational perspective, corneal MMP-14 modulation requires consideration of the delivery route, stromal penetration and residence time, safety tradeoffs related to wound healing, and target selectivity. Topical administration remains the most clinically attractive route due to its non-invasive nature, but rapid precorneal clearance and tear-film/epithelial barriers can limit stromal exposure, particularly for hydrophilic or high-molecular-weight agents and when sustained stromal levels are required [137]. Accordingly, preclinical ocular delivery studies often explore subconjunctival or intrastromal approaches to increase local stromal availability [138]. Subconjunctival delivery may improve exposure in the limbal region, whereas intrastromal delivery can provide higher spatial precision for localized disease, albeit with greater invasiveness and practical limitations for repeated dosing [139]. Because MMP-14 participates in physiological matrix remodeling and wound healing, excessive or prolonged inhibition may carry risks of delayed repair or altered tissue remodeling, supporting the need for dose optimization and temporal control [99]. Finally, selectivity is likely to influence tolerability, as broad-spectrum, Zn^2+^-chelating MMP inhibitors (including hydroxamate-based compounds) have been limited by off-target inhibition and musculoskeletal adverse effects, providing a rationale for more selective strategies when sustained modulation is contemplated [140].

### 6.2. MMP Inhibitors with Potential Use in CoNV

The trajectory of MMP inhibitor development provides critical insight for advancing MMP-14-targeted therapy in CoNV. Early first-generation hydroxamate inhibitors potently chelated catalytic zinc, but were non-selective, inhibiting dozens of MMPs and ADAM/ADAMTS proteases and causing musculoskeletal toxicity [141]. These failures stalled the field for more than a decade. Second-generation non-hydroxamate inhibitors improved tolerability but still lacked sufficient specificity or clinical efficacy [142].

A major shift occurred with structure-guided inhibitor design, which focuses on exploiting unique S1′ pocket geometry and surface exosite differences among MMPs [143]. This third generation has produced compounds with meaningful isoform selectivity, reviving interest in therapeutic MMP inhibition. Fourth-generation strategies now emphasize allosteric modulation, PEX-domain targeting, and protein-based inhibitors (engineered N-TIMP variants, monoclonal antibodies), which circumvent zinc chelation and significantly reduce off-target activity [144].

Despite a strong mechanistic rationale, MMP inhibitors have struggled to translate clinically. A key issue is that MMP functions are context- and stage-dependent, and broad inhibition can block enzymes with host-protective roles, which may compromise efficacy or even be harmful if the wrong target or timing is chosen [140]. Progress has also been limited by variable pharmacokinetics and a lack of practical biomarkers to confirm target engagement in the affected tissue [140,142]. Even selective agents such as the anti–MMP-14 antibody DX-2400 have largely remained preclinical, suggesting that specificity alone is not enough without clear proof-of-mechanism in the relevant disease setting [145].

Given the central role of MMP-14 in ECM degradation, pro-angiogenic signaling, and endothelial invasion, selective inhibition of this enzyme has emerged as an attractive therapeutic strategy for CoNV. Recent work has identified several classes of MMP inhibitors—ranging from repurposed drugs to engineered proteins and monoclonal antibodies—that demonstrate potential for targeting MMP-14 in ocular disease.

#### 6.2.1. Structural Basis of MMP-14 as a Therapeutic Target: Implications for Selective Inhibitor Development)

**Figure 4:**
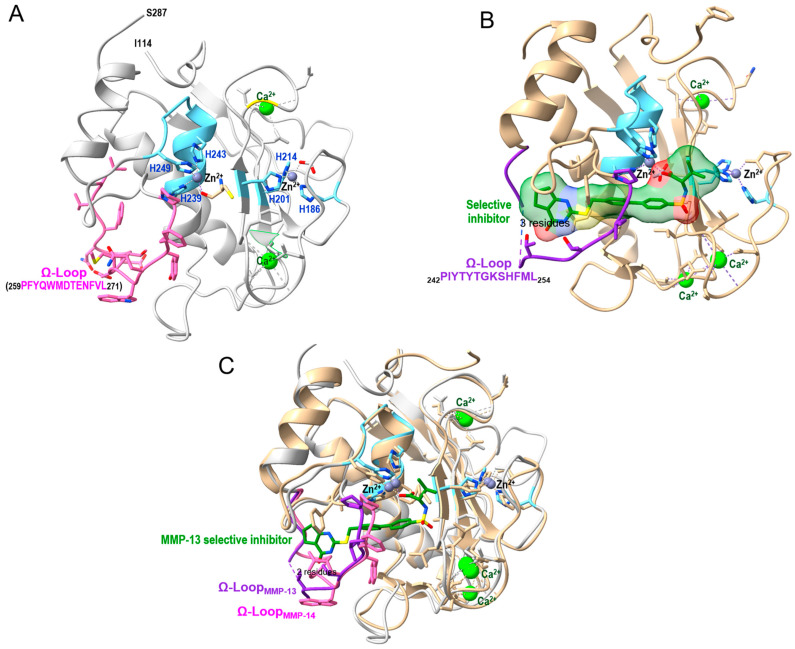
Structural comparison of MMP-14 (PDB: 1BQQ) and MMP-13 (PDB: 5UWK) highlighting Ω-loop differences and selective inhibitor binding. (A) Crystal structure of the MMP-14 (MT1-MMP) catalytic domain (PDB:1BQQ) [146]. The catalytic zinc ion (Zn^2+^) is shown as a gray sphere coordinated by His239, His243, and His249 (blue). Structural calcium ions (Ca^2+^) are shown as green spheres. The Ω-loop (residues 259–271; PFYQWMNTENFVL) is highlighted in magenta, forming part of the S1′ pocket wall and contributing to tunnel restriction. (B) Crystal structure of MMP-13 catalytic domain in complex with a selective inhibitor (PDB:5UWK) [147]. The inhibitor is shown in green sticks and occupies the deep S1′ pocket. The Ω-loop of MMP-13 (residues 242–254) is shown in purple. The catalytic Zn^2+^ ion is depicted as a gray sphere and Ca^2+^ ions as green spheres. Key active-site residues shaping the S1′ cavity are shown in stick representation. (C) Structural overlay of MMP-14 (1BQQ, gray) and MMP-13 (5UWK, tan) catalytic domains. The Ω-loop of MMP-13 is shown in purple and that of MMP-14 in magenta. The MMP-13 selective inhibitor (green) extends into the elongated S1′ pocket of MMP-13, whereas the corresponding region in MMP-14 is more restricted. Differences in Ω-loop positioning and side-chain orientation contribute to altered pocket depth and inhibitor selectivity between the two enzymes.

Matrix metalloproteinases (MMPs) share a conserved catalytic core characterized by the Zn^2+^-binding motif (HExGHxxGxxH) and a structurally stabilized β-sheet/α-helical fold. However, despite high conservation of the catalytic machinery, important structural differences exist within the S1′ substrate-binding pocket, particularly in the Ω-loop region, which critically influences inhibitor selectivity. The catalytic domain of MMP-14 (MT1-MMP; PDB:1BQQ) contains the canonical zinc-binding motif (His239, His243, His249) coordinating the catalytic Zn^2+^ ion (Figure 4A). The Ω-loop (residues 259–271; PFYQWMNTENFVL) forms a structural wall of the S1′ pocket. Notably, Trp263 and Met264 project into the pocket, creating a relatively restricted, medium-depth tunnel. This conformational constraint limits the accommodation of bulky P1′ substituents and distinguishes MMP-14 from collagenases such as MMP-13. In contrast, the Ω-loop of MMP-13 (PDB:5UWK; residues 244–255: PIYTYTGKSHFML) adopts a conformation that generates a deeper and more elongated S1′ cavity (Figure 4B). Tyr247 contributes to S1′ pocket plasticity by adopting alternative side-chain conformations across crystal structures, participating in induced-fit adjustments that modulate cavity depth and shape. The overlay shown in Figure 4C highlights that subtle backbone positioning differences in the Ω-loop, rather than alterations in the catalytic zinc site, primarily determine pocket geometry and ligand accommodation.

#### 6.2.2. Structural Basis of Current MMP Inhibitors

First-generation MMP inhibitors were predominantly hydroxamate-based zinc chelators. While these compounds effectively coordinate the catalytic Zn^2+^ ion, their reliance on metal chelation combined with the highly conserved active site resulted in poor isoform selectivity and dose-limiting musculoskeletal toxicity.

Second-generation inhibitors shifted toward non-hydroxamate zinc-binding groups or partial chelators, attempting to exploit subtle structural differences in the S1′ pocket. Structural studies (e.g., MMP-13 complexed with selective inhibitors in PDB:5UWK) demonstrate that selectivity arises not from differential Zn^2+^ coordination but from tailored interactions with Ω-loop residues lining the S1′ tunnel.

In MMP-13, the elongated S1′ pocket accommodates extended hydrophobic substituents, whereas in MMP-14, steric restriction imposed by Trp263 and Met264 narrows the distal region. Therefore, inhibitors designed for deep S1′ penetration in MMP-13 may be sterically incompatible with MMP-14.

#### 6.2.3. Prospects for Developing MMP-14–Specific Inhibitors

The development of MMP-14-specific inhibitors requires strategies that move beyond classical zinc-chelating approaches and instead exploit subtle structural features unique to this membrane-type MMP. Although the catalytic Zn^2+^ center is highly conserved across the MMP family, structural analyses reveal that the Ω-loop (residues 259–271) and the resulting S1′ pocket geometry in MMP-14 differ significantly from those of collagenases such as MMP-13. In particular, Trp262 and Met263 partially restrict the S1′ tunnel, creating a medium-depth, sterically constrained pocket that is less accommodating to elongated P1′ substituents. Rational inhibitor design should therefore focus on scaffolds optimized for this narrower topology, favoring precise shape complementarity rather than deep tunnel penetration. Additionally, targeting regions outside the conserved catalytic zinc site—including exosites, allosteric surfaces, or the hemopexin (HPX) domain—may enhance selectivity and reduce off-target inhibition. Given MMP-14′s membrane localization and central role in pericellular proteolysis, strategies incorporating spatial targeting, antibody-based approaches, or domain-selective inhibition may further improve therapeutic specificity. Collectively, structure-guided design that leverages Ω-loop architecture, pocket constraint, and domain-specific features offers a promising pathway toward selective MMP-14 modulation.

Although MMP catalytic cores are structurally conserved, differences in Ω-loop architecture and S1′ pocket geometry provide a mechanistic basis for isoform selectivity. Structural comparison between MMP-14 (1BQQ) and MMP-13 (5UWK) demonstrates that inhibitor specificity is dictated primarily by pocket depth, loop flexibility, and distal residue positioning rather than zinc coordination itself. These features underscore the feasibility—but also the complexity—of developing MMP-14–specific therapeutic agents.

#### 6.2.4. First-Generation Inhibitors (Broad-Spectrum/Zn^2+^ Chelators)

Hydroxamate inhibitors are the first generation of MMP inhibitors. They contain a hydroxamate zinc-binding group that has strong interaction with the catalytic domain of MMPs to inhibit cell growth, invasion, and metastasis. The most common hydroxamate-based inhibitors are Batimastat and Marimastat [144]. Batimastat is effective in blocking MMP-1, MMP-2, MMP-7, MMP-8, MMP-9, and MMP-14 [148]. Batimastat entered Phase 1 clinical trials as a cancer therapy in the late 1990s. It was shown to inhibit at least 50% of MMP activity but was poorly soluble, making its pharmacokinetic profile less suitable for humans [149]. Marimastat is closely related to Batimastat, retaining its efficacy in MMP inhibition of MMP-2, MMP-9, MMP-7, and MMP-14, but with a better oral bioavailability [150]. Marimastat entered clinical trials as a cancer therapy in the late 1990s and continued through the early 2000s [150,151].

While these hydroxamates are promising MMP inhibitors, there is consistent development of musculoskeletal toxicity with their usage. Rats treated with Marimastat for two weeks via subcutaneous pumps all showed various clinical signs of musculoskeletal degradation, including inability to move, high-stepping gait, and synovial hyperplasia [141]. The side effect also persists in human pharmacologic usage of hydroxamate. Reversible arthralgia, stiffness, and myalgia occurred in patients after 12-month administration of PG-11680, another hydroxamate-based MMP inhibitor, for osteoarthritis [152]. In addition to the major musculoskeletal side effects, the hydroxamates also have short half-lives, requiring constant injections and limiting their clinical applications [141].

#### 6.2.5. Second-Generation Inhibitors (Selective)

Non-hydroxamate-based inhibitors weakly chelate the zinc binding group of MMPs with chemical groups including, but not limited to, carboxylic acids, thiols, sulfonyl hydrazides, or aminomethyl benzimidazole [142]. Many of these second-generation inhibitors entered clinical trials for anti-malignant therapy in the early 2000s. They effectively inhibit MMPs while having decreased rates of musculoskeletal syndrome compared to the first-generation [142,144]. But the therapeutic impacts were minimal or still induced unacceptable levels of toxicity, making them unfit candidates for possible treatments of CoNV [153,154]. However, the non-hydroxamate antibiotic derivative MMP inhibitors are promising as second-generation MMP inhibitor treatments for CoNV.

Tanomastat (BAY 12-9566) is a biphenyl compound, containing a thioether zinc-binding group, that was entered Phase III clinical trials for various cancers, including small cell lung cancer and advanced ovarian cancer, in the early 2000s [153,155]. It inhibits MMPs-2, -3, -9, -13, and -14 [155,156]. While Tanomastat was well-tolerated in patients, there was no evidence of an impact on survival related to ovarian cancer [153].

Rebimastat (BMS-275291) contains a thiol and mercaptoacyl zinc-binding group that entered clinical trials for non-small-cell lung cancer and Kaposi sarcoma [154,157,158]. Similar to Tanomastat, Rebimastat inhibits MMPs-2, -3, -9, -13, and -14 [159]. In mouse models of experimental lung metastases, Rebimastat inhibited angiogenesis and endothelial cell migration [159]. However, this drug was not viable in humans, as in both clinical trials, Rebimastat induced unacceptable toxicity in patients and did not improve the chances of survival [154,157].

Doxycycline, a semi-synthetic tetracycline, inhibits MMPs noncompetitively by chelating catalytic Zn^2+^ [160]. Although not selective for MMP-14, it reduces angiogenesis through both MMP-dependent and MMP-independent mechanisms. Animal studies and a clinical pilot trial showed that topical 1% doxycycline decreased neovascular area and vessel caliber in five out of six CoNV [161,162,163].

Metastat (COL-3) is a tetracycline-based inhibitor that targets MMP-2, -9, and -14 by decreasing trypsinogen-2 and nitric oxide, which are known regulators of MMP activity [164,165]. In a study conducted by Lokeshwar et al. [164], rats with metastatic prostate cancer treated with Metastat showed significant decreases in MMP-2, MMP-14, and TIMP-2 compared to those treated with doxycycline. There were significant decreases in tumor size, metastasis, and delays in tumor growth, accompanied by limited toxicity to the rats [164]. Due to its specific targeting of MMP-14 and its close relationship to Doxycycline, this antibiotic could prove promising for reducing CoNV and warrants further exploration.

Clioquinol and Chloroxine, clinically used antibacterial and antifungal quinoline derivatives, were recently identified as selective binders of the catalytic domain of MMP-14, demonstrating inhibition of enzyme activity [166]. Clioquinol did not significantly inhibit MMP-2, MMP-7, MMP-9, or MMP-13, indicating high selectivity for MMP-14 among the MMP family. Additionally, significantly reduced activation of pro-MMP-2 in the cell was observed due to the inhibition of MMP-14 by these compounds [166]. Because these agents are already used clinically, they may serve as promising lead compounds for the development of MMP-14–targeted therapies in angiogenic eye diseases.

#### 6.2.6. Third Generation Inhibitors (Structure-Guided/Catalytic-Site Selective)

Third-generation MMP inhibitors target recognition pockets of the catalytic domain of MMPs that flank the zinc-binding group [144]. The left-hand side of the zinc pocket is “unprimed,” and the right-hand side is “primed”, designated as S3, S2, S1, and S3′, S2′, S1′ respectively. Of these pockets, the S1′ pocket varies the most amongst different MMPs, which makes it the best target for selective MMP inhibitors [167]. There are three pocket depths for S1′: shallow, intermediate, and deep. While previous papers have stated that MMP-14 has a deep pocket, more recent research has shown that the pocket depth is intermediate [143,166]. Therefore, it holds similar depth to MMP-2, MMP-8, MMP-9, and MMP-26 [32,144,167]. MMP-14 has the same length of Ω-loop as MMP-8 and MMP-13, with a unique M264 residue, providing further selectivity for MMP-14 [166].

(R)-ND-336, a highly selective inhibitor for MMP-9, has also been shown to inhibit MMP-14 in cell migration and collagen contraction assays. (R)-ND-336 attenuated human conjunctiva fibroblast migration and mitigated collagen contraction, thus highlighting its potential as a therapeutic resource for fibroproliferative ocular diseases, such as pterygium [168].

SB-3CT is another well-studied third-generation inhibitor of MMP-9 and MMP-2 that has been shown to reduce corneal neovascularization. Mice given SB-3CT eye drops twice daily for a week had significantly reduced corneal lymphangiogenesis and macrophage infiltration [169]. SB-3CT has been shown to be a linear competitive inhibitor of MMP-14; however, the residence time is less than 1 s [170]. The search for a third-generation MMP-14 inhibitor has been challenging because the S1′ site is shallower and less flexible than those of other MMPs. However, a promising avenue for creating a third-generation MMP-14 inhibitor is to modify the current highly selective MMP-2 or MMP-9 inhibitors.

#### 6.2.7. Fourth Generation Inhibitors (Allosteric, Exosite, or Protein-Based MMP-14 Blockers) Engineered N-TIMP-2 Variants

Bonadio and colleagues developed a highly selective inhibitor of MMP-14 using a computational protein engineering approach based on N-TIMP-2 [171]. The researchers modified specific loops in N-TIMP-2. The resulting molecule exhibited nanomolar affinity for MMP-14 and over 1000-fold selectivity relative to other MMPs [171]. This method reduces unwanted effects on other proteins and may be a more accurate way to block MMP-14.

#### 6.2.8. Monoclonal Antibodies Targeting MMP-14

A recombinant antibody fragment Fab-3A2 has shown specific effects targeting MMP-14 to reduce cancer metastasis through competitive inhibition to prevent MMP-14 from activating pro-MMP-2. IgG 3A2 effectively reduced cancer spread, improved immune responses, and targeted cancer stem cells, with better stability and fewer dosing requirements than the Fab form in a melanoma xenograft mouse model. Unlike broad-spectrum MMP inhibitors, IgG 3A2 is highly selective and shows improved potency (IC_50_ 3.8 nM) compared to other inhibitors [172]. Several monoclonal antibodies used to inhibit MMP-14 in tumors may also have potential applications in future CoNV treatments. Notable examples include Dx-2400, 9E8, R2C7, and 3369, which have been found to reduce angiogenesis, delay lymphogenesis, target the MMP-14 catalytic domain, and block extracellular matrix degradation, respectively, across various cancers [124,173,174,175,176]. While these monoclonal antibodies show promise in enhanced selectivity for MMP-14, they are largely still in the preclinical stage.

#### 6.2.9. PEX-Domain-Targeting Compounds

NSC-405020 (3,4-dichloro-N-(pentan-2-yl)benzamide) has also been found to electrochemically target the conserved PEX domain of MMP-14. Although it has primarily been used as a probe for MMP-14 detection, in the future, it could potentially be paired with an inhibitor to selectively target and inhibit MMP-14 [83,177].

#### 6.2.10. Other Potential MMP-14 Modulators (Natural Products & Multifunctional Agents)

Natural products are generally considered more biocompatible and less toxic than synthetic MMP inhibitors, making them attractive therapeutic leads; however, their mechanistic ambiguity remains a major challenge. While many polyphenols and phytochemicals attenuate CoNV, their anti-angiogenic effects are typically broad and non-selective, and the extent to which they directly modulate MMP-14 or other metalloproteinases remains unclear. For example, Naringenin, a type of polyphenol (i.e., flavanone) found in citrus fruits and vegetables, has anti-inflammatory and anti-angiogenic properties [178]. In a murine alkali burn model, naringenin reduced CoNV and decreased MMP-14 mRNA expression. This inhibition of MMP-14, along with its effects on inflammatory cytokines and pro-angiogenic factors, points to naringenin as a potential small molecule that modulates MMP-14, contributing to its therapeutic effects in reducing angiogenesis and inflammation [179].

Curcumin, also known as turmeric, is a natural product that has also been shown to inhibit MMP-14. In a paper by Wang et al. [180], curcumin was shown to have a binding site in MMP-14. When bladder cancer cells were exposed to curcumin, their ability to proliferate and migrate was significantly diminished. Through a Western blot including MMP-14, the researchers discovered that curcumin specifically inhibited the AKT/MMP-14 pathway [180]. While CoNV happens in a different organ, AKT pathway activation by VEGFA is a major pathway leading to pathogenesis [98]. Therefore, curcumin or derivative compounds could potentially inhibit MMP-14′s role in CoNV.

While MMP-14-targeted probes have primarily been developed to monitor enzyme activity and spatial distribution, their increasing use in clinical studies underscores the growing translational relevance of MMP-14. Ongoing trials across diverse disease contexts have generated valuable data on dosing, specificity, and off-target effects that may inform ocular applications. Importantly, insights into delivery strategies and safety profiles from these studies could accelerate the development of MMP-14–directed therapies for corneal neovascularization and other forms of pathological angiogenesis.

## 7. Methods for Literature Review

We performed a targeted literature search to support this narrative review using PubMed, Scopus, and Web of Science, covering the literature from the earliest records available through January 2026. Search terms were centered on “MMP-14” and “MT1-MMP” and combined with cornea- and mechanism-focused keywords, including “corneal neovascularization,” “corneal angiogenesis,” “lymphangiogenesis,” “VEGFR1,” “inflammation,” “trafficking,” and “regulation.” We prioritized peer-reviewed studies directly focused on corneal physiology and pathology. Selected non-ocular literature was included only when it provided mechanistic insight into MMP-14 regulation, intracellular trafficking, substrate or receptor processing, or inhibition strategies that were directly relevant to the corneal context discussed here. Articles not addressing MMP-14 specifically or outside the scope of this review were excluded. Reference lists of key articles were also screened to identify additional relevant studies.

## 8. Conclusions

MMP-14 is a central regulator of corneal neovascularization, a complex but ordered and step-dependent process [10]. By cleaving several different substrates, MMP-14 remodels the surrounding ECM, acts on growth-factor signaling, and modulates VECs migration and invasion, contributing to how the cornea loses or maintains its angiogenic privilege [7]. Its functions are multifaceted and depend on the local context.

Through the degradation of type I collagen and the activation of pro-MMP-2, MMP-14 allows VECs to migrate and organize in tubules to form new vessels [5]. It also coordinates VEGF-driven angiogenesis by upregulating VEGF expression [3] and modulating VEGF receptor signaling [100]. In contrast, MMP-14 can exert anti-angiogenic effects by cleaving VEGFR1, leading to a VEGF-trap mechanism [96] and by processing collagen XVIII to generate endostatin and neostatin-14, which inhibit angiogenesis [101]. Moreover, after corneal injury, MMP-14 expression rises and contributes to tissue repair by reorganizing the stroma, activating MMP-2, and reducing fibrotic scarring. Experimental overexpression has been shown to improve corneal transparency and downregulate markers of fibrosis, such as α-SMA and type III collagen [111].

Clinically, the available treatments for CoNV (mainly corticosteroids, immunosuppressive medications, and anti-VEGF agents) may provide only partial and temporary control and have frequent side effects [127]. Targeting MMP-14 represents a potential alternative or complementary approach. Current strategies include small-molecule inhibitors [166], tetracyclines [163], selective TIMP-2 variants [171], recombinant antibodies [172], and natural compounds like naringenin [179].

Future studies should explore the molecular and contextual factors that determine whether MMP-14 functions as a pro- or anti-angiogenic factor in the cornea. The interactions of MMP-14 with other molecules, such as integrins, TIMPs, and receptor tyrosine kinases, likely help regulate the delicate balance between vascular growth and inhibition. Translational research studies should assess the efficacy and safety of selective MMP-14 inhibitors in comparison to current therapies. Ultimately, the goal of elucidating the factors and signaling pathways that regulate MMP-14 production and function is to provide targeted inhibition of MMP-14-mediated corneal angiogenesis, thereby maintaining visual clarity after injury or inflammation.

## Figures and Tables

**Table 1:** Evidence map of MMP-14-related angiogenic mechanisms discussed in Section 4. The table summarizes, for each mechanism, the experimental system (in vitro/ex vivo/in vivo), primary cell type, angiogenic directionality (pro- or anti-angiogenic, or not assessed), and whether the evidence is cornea-specific.

Mechanism	Experimental System	Cell type	Outcome Direction	Cornea- Specific?
VEGFR1 cleavage leading to “VEGF-trap” fragment [96]	In vitro	Mouse corneal fibroblasts; Calf pulmonary VECs	Anti-angiogenic	Partial
Exosome-associated VEGFR1 cleavage [97]	In vitro	Mouse corneal fibroblasts; HUVECs	Pro-angiogenic	Partial
Collagen XVIII cleavage generating neostatin-14 [101]	In vitro, In vivo (mouse cornea, bFGF-induced micropocket NV)	Calf pulmonary VECs; mouse cornea	Anti-angiogenic	Yes
EphA2 cleavage/truncation [107]	In vitro, ex vivo	Human cancer cells; Human ovarian adenocardinomas	Pro-oncogenic	No
Collagen I proteolysis (vessel sprouting, lumen formation)	3D collagen I matrices (ex vivo [5]; in vitro [112])	VECs	Pro-angiogenic	No
MMP-14-dependent FGF-2-induced CoNV [118]	In vivo (mouse cornea)	Mouse cornea (tissue-level response)	Pro-angiogenic (required)	Yes
MMP-14-PDGF receptor-β signaling (mural cell investment) [120]	In vitro, in vivo (mouse)	Mouse vascular smooth muscle cells, pericytes	Pro-angiogenic (vessel maturation/stabilization)	No
Endoglin shedding [2]	In vitro, ex vivo	HUVECs, ECRF	Anti-angiogenic	No
Protease-independent epithelial anti-angiogenic effect [123]	In vitro	Mouse corneal epithelial cells, calf pulmonary VECs	Anti-angiogenic	Yes
Decorin cleavage [102]	In vitro, in vivo (mouse cornea)	Corneal stromal cells, VECs	Pro-angiogenic	Yes

## Data Availability

No new data were created or analyzed in this study.

## Supplementary Materials

The following supporting information can be downloaded at https://www.mdpi.com/article/10.3390/ijms27042027/s1. References [181,182,183,184,185,186,187,188,189,190,191,192,193,194,195,196,197,198,199,200,201,202,203,204,205,206,207,208,209,210,211,212,213,214,215,216,217,218,219,220] are cited in supplementary file.

## Abbreviations

The following abbreviations are used in this manuscript:

AMD	Age-Related Macular Degeneration
bFGF	Basic fibroblast growth factor
CoNV	Corneal neovascularization
COPD	Chronic Obstructive Pulmonary Disease
DNA	Deoxyribonucleic acid
EphA2	Ephrin type-A receptor 2
EGF	Epidermal growth factor
ECM	Extracellular matrix
ERK	Extracellular signal-regulated kinase
Flk-1	Fetal liver kinase 1
GPI	Glycosylphosphatidylinositol
HGF	Hepatocyte growth factor
HIF-1	Hypoxia-inducible factor 1
HUVECs	Human umbilical vein endothelial cells
IL-1ß	Interleukin-1 beta
Ilomastat-CD	Ilomastat-cyclodextrin
LYVE-1	lymphatic vessel endothelial hyaluronan receptor 1
mRNA	Messenger ribonucleic acid
MMP	Matrix metalloproteinase
PLGF	Placental growth factor
PDGF	Platelet-derived growth factor
SMCs	Smooth muscle cells
Src	Proto-oncogene tyrosine-protein kinase Src
TIMPs	Tissue inhibitors of metalloproteinases
TGF	Transforming growth factor
TLR	Toll-like receptor
TNF-α	Tumor necrosis factor alpfa
VECs	Vascular endothelial cells
VEGF	Vascular endothelial growth factor
VEGFR	Vascular endothelial growth factor receptor
