# Supplementary material for: Matrix Metalloproteinase 14 in Corneal Neovascularization
Source: Int J Mol Sci. 2026 Feb 20;27(4):2027. doi: 10.3390/ijms27042027 (PMC12940456; doi:10.3390/ijms27042027)
Supplement: Supplementary file 1 [file ijms-27-02027-s001.zip › ijms-4129913-supplementary.pdf]

International Journal of *Molecular Sciences*

MDPI

## Supplementary Materials

**Table S1.** This table identifies various MMPs and their biological significance. Notably, it encompasses their distribution in the body, key substrates, physiological process involvement, and pathological condition involvement. Adapted from Cui et al., 2017 [1].

| MMP | Chromosome Location | Distribution | Key Substrates | Physiological Process Involvement | Pathological Condition Involvement |
|---|---|---|---|---|---|
| Collagenases | | | | | |
| **MMP-1** [18,181-183] | 11q22.3 | Endothelium, fibroblasts, intima, platelets, SMCs, varicose veins, vascular adventitia | Aggrecan, nidogen, perlecan, proteoglycan link protein, serpins, tenascin-C, versican, casein, $\alpha$1-antichymotrypsin, $\alpha$1-antitrypsin, $\alpha$1-proteinase inhibitor, IGF-BP-3 and -5, IL-1β, L-selectin, ovostatin, pro-TNF-$\alpha$, SDF-1, various collagen substrates, gelatin | Immune response, wound healing | Fibrotic disorder (lung fibrosis, liver fibrosis), inflammation cytokines, cardiovascular (atherosclerosis, aneurysm, MI), cancer (nasopharyngeal, esophageal, colorectal, breast), venous hypertension, carotid atheroma, insulin resistance, eye cancers (uveal melanoma, retinoblastoma), primary open-angle glaucoma [18] |
| **MMP-8** [182-185] | 11q22.3 | Macrophages, neutrophils | Aggrecan, elastin, fibronectin, laminin, nidogen, $\alpha$2-antiplasmin, proMMP-8, various collagen substrates, gelatin | Wound healing | Inflammatory cytokines, unstable carotid atheroma |
| **MMP-13** [182,186,187] | 11q22.3 | Breast cancer, macrophages, preeclampsia, SMCs, varicose veins | Aggrecan, fibronectin, laminin, perlecan, tenascin, casein, plasminogen activator 2, proMMP-9 and -13, SDF-1, various collagen substrates, gelatin | Immune response, tissue remodeling | Inflammation cytokines, lung disease (asthma, COPD), osteoarthritis, viral infection (adenovirus, influenza), cancer (nasopharyngeal, esophageal, colorectal, breast), neointima formation, restenosis/atherothrombosis, renal disease |

 https://doi.org/10.3390/ijms27042027

| | | | | | |
|---|---|---|---|---|---|
| **MMP-18** [188] | 12q14 | Xenopus (amphibian) colon, heart, lung | $\alpha$1-antitrypsin, various collagen substrates, gelatin | Axonal growth | Unknown |
| Gelatinases | | | | | |
| **MMP-2** [16,18,181,182,185,189-194] | 16q13-q21 | Adventitia, aortic aneurysm, endothelium, leukocytes, platelets, varicose veins, VSM | Aggrecan, elastin, fibronectin, laminin, nidogen, proteoglycan link protein, versican, active MMP-9 and -13, FGF-R1, IGF-BP-3 and -5, IL-1β, pro-TNF-$\alpha$, TGF-β, various collagen substrates, gelatin | Angiogenesis | Chronic venous disease (varicose veins, venous leg ulcer), inflammation cytokines, viral infection (adenovirus, influenza), cardiovascular (atherosclerosis, aneurysm, MI), cancer (nasopharyngeal, esophageal, colorectal, breast), ICAS, thrombosis, heart and renal disease, eye cancers (uveal melanoma, retinoblastoma), eye disorders (diabetic retinopathy, dry eye, cataracts, primary open angle glaucoma) |
| **MMP-9** [16,18,181,182,185,189-194] | 20q11.2-q13.1 | Adventitia, aortic aneurysm, endothelium, macrophages, microvessels, varicose veins, VSM | Aggrecan, elastin, fibronectin, laminin, nidogen, proteoglycan link protein, versican, CXCL5, IL-1β, IL2-R, plasminogen, pro-TNF-$\alpha$, SDF-1, TGF-β, various collagen substrates, gelatin [28] | Cell apoptosis | Chronic venous disease (varicose veins, venous leg ulcer), osteoarthritis, cardiovascular (atherosclerosis, aneurysm, MI), cancer (nasopharyngeal, esophageal, colorectal, breast), hypertension, carotid stenosis, cardiac and renal disease, eye cancers (uveal melanoma, retinoblastoma), eye disorders (diabetic retinopathy, dry eye, cataracts, primary open angle glaucoma) |

https://doi.org/10.3390/ijms27042027

| | | | | | |
|---|---|---|---|---|---|
| Stromelysins | | | | | |
| **MMP-3** [16,18,181,182,189,191,193] | 11q22.3 | Coronary artery disease, endothelium, hypertension, intima, platelets, synovial fibroblasts, tumor invasion, varicose veins, VSM | Aggrecan, decorin, elastin, fibronectin, laminin, nidogen, perlecan, proteoglycan, proteoglycan link protein, versican, casein, $\alpha$1-antichymotrypsin, $\alpha$1-proteinase inhibitor, antithrombin III, E-cadherin, fibrinogen, IGF-BP-3, L-selectin, ovostatin, pro-HB-EGF, pro-IL-1β, proMMP-1, -8 and -9, pro-TNF-$\alpha$, SDF-1, various collagen substrates, gelatin | Tissue remodeling | Chronic venous disease (varicose veins, venous leg ulcer), fibrotic disorder (lung fibrosis, liver fibrosis), liver disease (cirrhosis, portal hypertension), osteoarthritis, cardiovascular (atherosclerosis, aneurysm, MI), cancer (nasopharyngeal, esophageal, colorectal, breast), IBD, eye disorders (dry eye syndrome, primary open-angle glaucoma) |
| **MMP-10** [182,189,195-197] | 11q22.3 | Arthritis, atherosclerosis, carcinoma cells, preeclampsia, uterus | Aggrecan, elastin, fibronectin, laminin, nidogen, casein, proMMP-1, -8 and -10, various collagen substrates, gelatin | Cellular apoptosis, tissue remodeling, wound healing | Chronic venous disease (varicose veins, venous leg ulcer), liver disease (cirrhosis, portal hypertension), lung disease (asthma, COPD), viral infection (adenovirus, influenza), cancer (nasopharyngeal, esophageal, colorectal, breast), IBD, microvascular disease in diabetes, muscular dystrophy |
| **MMP-11** [198,199] | 22q11.23 | Angiogenesis, brain, uterus | Aggrecan, fibronectin, laminin, $\alpha$1-antitrypsin, $\alpha$1-proteinase inhibitor, IGF-BP-1 | Wound healing, mammary gland development [18] | Cancer (nasopharyngeal, esophageal, colorectal, breast) |

https://doi.org/10.3390/ijms27042027

| | | | | | |
|---|---|---|---|---|---|
| Matrilysins | | | | | |
| **MMP-7** [16,18,181,182,200,201] | 11q21-q22 | Endothelium, intima, uterus, varicose veins, VSM | Aggrecan, elastin, enactin, fibronectin, laminin, proteoglycan link protein, casein, β4 integrin, decorin, defensin, E-cadherin, Fas-ligand, plasminogen, proMMP-2, -7, and -8, [190] pro-TNF-α, syndecan, transferrin, various collagen substrates, gelatin [182] | Cellular apoptosis | Inflammatory cytokines, lung disease (asthma, COPD), cardiovascular (atherosclerosis, aneurysm, MI), cancer (nasopharyngeal, esophageal, colorectal, breast, acute myeloid leukemia), Kawasaki disease, coronary artery disease, and hypertension |
| **MMP-26** [202,203] | 11p15 | Breast cancer, endometrial tumors | Fibrinogen, fibronectin, vitronectin, casein, β1-proteinase inhibitor, fibrin, fibronectin, proMMP-2, collagen IV, gelatin | Tissue remodeling, wound healing | Chronic venous disease (varicose veins, venous leg ulcer), cancer (nasopharyngeal, esophageal, colorectal, breast), cerebral amyloid angiopathy |
| Membrane-Type | | | | | |
| **MMP-14** [18,203] | 14q11-q12 | Angiogenesis, brain, fibroblasts, platelets, uterus, VSM | Aggrecan, elastin, fibrin, fibronectin, laminin, nidogen, perlecan, proteoglycan, tenascin, vitronectin, αvβ3 integrin, CD44, proMMP-2 and -13, pro-TNF-α, SDF-1, α1-proteinase inhibitor, tissue transglutaminase, various collagen substrates, gelatin | Cellular apoptosis, immune response, morphogenesis, tissue remodeling | Cardiovascular (atherosclerosis, aneurysm, MI), cancer (nasopharyngeal, esophageal, colorectal, breast, lung, bladder), neuroinflammation, neointima formation, pressure overload, macular degeneration, and hypertension |
| **MMP-15** [201,204,205] | 16q13 | Fibroblasts, leukocytes, and preeclampsia | Aggrecan, fibronectin, laminin, nidogen, perlecan, tenascin, vitronectin, ProMMP-2 and -13, tissue transglutaminase, collagen I, gelatin | Morphogenesis[18] | Cancer (nasopharyngeal, esophageal, colorectal, acute myeloid leukemia), inflammatory disease |

https://doi.org/10.3390/ijms27042027

| | | | | | |
|---|---|---|---|---|---|
| **MMP-16** [18,206,207] | 8q21.3 | Angiogenesis, leukocytes | Aggrecan, fibronectin, laminin, perlecan, vitronectin, casein, proMMP-2 and -13, collagen I | Angiogenesis[18] | Cancer (nasopharyngeal, esophageal, colorectal, breast, prostate), HSP, and DVT |
| **MMP-17** [208,209] | 12q24.3 | Brain, breast cancer | Fibrin, gelatin | Unknown | Cancer (nasopharyngeal, esophageal, colorectal, breast), aortic aneurysm |
| **MMP-24** [205,208] | 20q11.2 | Astrocytoma, brain, glioblastoma, kidney, leukocytes, lung, pancreas | Chondroitin sulfate, dermatin sulfate, fibrin, fibronectin, N-cadherin, ProMMP-2 and -13, gelatin | Immune response | Neurological disease (neuropathic pain, neural plasticity), cancer (nasopharyngeal, esophageal, colorectal, breast), atherosclerosis |
| **MMP-25** [210] | 16p13.3 | Anaplastic astrocytomas, glioblastomas, leukocytes | Fibrin, fibronectin, proMMP-2, $\alpha$1-proteinase inhibitor, collagen IV, gelatin | Cellular apoptosis, immune response | Lung disease (asthma, COPD), cancer (nasopharyngeal, esophageal, colorectal, breast), abdominal aortic aneurysm |
| Other MMPs | | | | | |
| **MMP-12** [16,18,182,189,190,192-194] | 11q22.3 | Fibroblasts, great saphenous vein, macrophages, SMCs | Elastin, fibronectin, laminin, casein, plasminogen, collagen IV, gelatin | Lung disease (asthma, COPD), neurological disease (neuropathic pain, neural plasticity) | Lung disease (asthma, COPD), neurological disease (neuropathic pain, neural plasticity), viral infection (adenovirus, influenza), cancer (nasopharyngeal, esophageal, colorectal, breast), atherosclerosis, aortic dissection, retinopathy, intracerebral hemorrhage, peripheral vascular damage, DVT |
| **MMP-19** [18,203,204] | 12q14 | Liver | Aggrecan, fibronectin, laminin, nidogen, tenascin, casein, collagen I and IV, gelatin | Wound healing, angiogenesis [18] | Liver disease (cirrhosis, portal hypertension), lung disease (asthma, COPD), neurological disease (neuropathic pain, neural plasticity), osteoarthritis and RA, cancer (nasopharyngeal, esophageal, colorectal, breast), thoracic aortic aneurysms, cerebral amyloid angiopathy |
| **MMP-20** [211,212] | 11q22.3 | Tooth enamel | Aggrecan, cartilage oligomeric protein, amelogenin, collagen V | Tooth enamel formation | Enamel erosion, lung adenocarcinoma |

| | | | | | |
|---|---|---|---|---|---|
| **MMP-21** [202,213,214] | 10q26.13 | Fibroblasts, macrophages, and placenta | $\alpha$1-antitrypsin | Embryogenesis, tissue remodeling | Cancer (nasopharyngeal, esophageal, colorectal, breast, melanoma) |
| **MMP-22** [215] | 1p36.3 | Chicken fibroblasts | Gelatin | Tissue remodeling | Unknown; no data exists for humans. The human ortholog is MMP-27. |
| **MMP-23** [216] | 1p36.3 | Other (type II) MT-MMP, ovary, prostate, testis | Gelatin | Cellular apoptosis, reproduction, and menstruation [18] | Cancer (nasopharyngeal, esophageal, colorectal, breast), thrombosis |
| **MMP-27** [215,217] | 11q24 | Bone, breast cancer, endometrium, heart, kidney, leukocytes, macrophages, menstruation, osteoarthritis | Unknown | Embryogenesis, reproduction, and menstruation | Cancer (nasopharyngeal, esophageal, colorectal, breast) |
| **MMP-28** [218-220] | 17q21.1 | Keratinocytes, skin | Casein | Embryogenesis, tissue remodeling | Cardiovascular (atherosclerosis, aneurysm, MI), cancer (nasopharyngeal, esophageal, colorectal, breast, hepatocellular carcinoma), left ventricular remodeling, soft tissue edema, idiopathic pulmonary fibrosis |

https://doi.org/10.3390/ijms27042027
